# Adherence of Oncologists and Cardiologists to Venous Thromboembolic Disease Prevention and Treatment Guidelines in Cancer Patients: A Cross-Sectional Survey from Turkey

**DOI:** 10.3390/jcm15124504

**Published:** 2026-06-10

**Authors:** Ugur Onsel Turk, Mehmet Emin Arayici, Umut Kocabas, Kivanc Yuksel, Yasemin Basbinar, Hulya Ellidokuz

**Affiliations:** 1 Department of Cardiology, Medical Point Hospital, Faculty of Medicine, İzmir University of Economics, 35575 İzmir, Türkiye; umutkocabas@hotmail.com; 2 Department of Biostatistics and Medical Informatics, Faculty of Medicine, Dokuz Eylul University, 35330 İzmir, Türkiye; mehmet.e.arayici@gmail.com; 3 Department of Biostatistics and Medical Informatics, Faculty of Medicine, Ege University, 35100 İzmir, Türkiye; kivanc.yuksel@ege.edu.tr; 4 Department of Translational Oncology, Institute of Oncology, Dokuz Eylul University, 35330 İzmir, Türkiye; ybaskin65@gmail.com; 5 Department of Preventive Oncology, Institute of Oncology, Dokuz Eylul University, 35330 İzmir, Türkiye; hulyazeyda@gmail.com

**Keywords:** cancer-associated thrombosis, venous thromboembolism, clinical practice guidelines, anticoagulant therapy, risk stratification, guideline adherence, cardio-oncology

## Abstract

**Background:** Cancer-associated thrombosis (CAT) is a leading cause of morbidity and mortality in cancer patients. Although international guidelines provide comprehensive recommendations for venous thromboembolism (VTE) prevention and treatment, the degree to which clinicians adhere to these guidelines in routine practice remains unclear, particularly in countries with limited national data such as Turkey. **Methods:** A cross-sectional, descriptive survey was conducted among oncology specialists (medical oncologists, radiation oncologists, and surgical oncologists) and cardiologists practicing across Turkey. A structured, case-based questionnaire comprising 21 multiple-choice questions was distributed electronically via SurveyMonkey. The questionnaire assessed perioperative VTE prophylaxis approaches, VTE risk assessment practices in ambulatory patients, primary and long-term secondary thromboprophylaxis preferences, acute VTE treatment strategies, and management of special clinical scenarios. Responses were analyzed using descriptive statistics and compared between oncologist and cardiologist groups. **Results:** A total of 84 physicians participated (34 oncologists [40.5%], 50 cardiologists [59.5%]). Perioperative and inpatient VTE prophylaxis practices were largely concordant with guideline recommendations, with 67.9% individualizing prophylaxis decisions and 66.7% initiating prophylaxis in hospitalized immobile patients when not contraindicated. However, only 33.7% routinely performed VTE risk assessment in ambulatory patients, and 64.6% did not use any validated risk scoring system. Low-molecular-weight heparin (LMWH) was the preferred agent for acute VTE treatment (72.6%), while direct oral anticoagulants (DOACs) gained preference in long-term secondary thromboprophylaxis (42.2%). No statistically significant differences were observed between oncologists and cardiologists across all survey items (all *p* > 0.05). Notably, 94.1% of respondents expressed a need to update their knowledge regarding CAT management. **Conclusions:** While oncologists and cardiologists in Turkey demonstrate general awareness of CAT guidelines, significant gaps persist in VTE risk stratification and primary prophylaxis for ambulatory cancer patients. The near-universal self-reported need for knowledge updates highlights the urgency for structured multidisciplinary education programs, integration of validated risk scoring tools into clinical workflows, and development of nationally adapted clinical practice guidelines. These findings reflect self-reported practices and may not fully represent actual clinical behavior; future studies incorporating medical record reviews or prescription data are needed to validate these observations.

## 1. Introduction

Cancer-associated thrombosis (CAT) represents one of the most clinically significant complications encountered in oncology practice. Venous thromboembolism (VTE), encompassing deep vein thrombosis (DVT) and pulmonary embolism (PE), is the second leading cause of death in cancer patients and constitutes a major source of preventable morbidity and mortality [1,2]. Cancer patients face a four- to seven-fold increased VTE risk compared to the general population due to the interplay between malignancy-driven hypercoagulability, endothelial dysfunction, and venous stasis [3,4]. Approximately 20% of all VTE events occur in cancer patients, contributing to prolonged hospitalization, treatment delays, and long-term sequelae including post-thrombotic syndrome and chronic thromboembolic pulmonary hypertension [5,6,7,8,9,10]. Over the past decade, pivotal randomized controlled trials, including the CASSINI and AVERT studies, have established the role of direct oral anticoagulants (DOACs) in both primary prophylaxis and treatment of cancer-associated VTE [11,12], and major international clinical practice guidelines now provide detailed, evidence-based recommendations for VTE prevention and treatment in cancer patients [13,14,15,16,17,18].

Despite the availability of these comprehensive guidelines, a persistent gap exists between evidence-based recommendations and real-world clinical practice. Studies from various countries have demonstrated variable adherence to guideline recommendations, with particular deficiencies in ambulatory VTE risk assessment, systematic use of validated risk scoring tools such as the Khorana score, and appropriate selection of anticoagulant agents [19,20]. This guideline–practice gap is particularly concerning in countries where national-level data on CAT epidemiology and management are scarce. Turkey presents a compelling case for such an investigation. Despite having a substantial oncology patient population and an evolving cardio-oncology landscape, Turkey-specific data on CAT incidence, management patterns, and guideline adherence remain exceedingly limited [21,22]. The few available studies have been predominantly single-center, retrospective in nature, and restricted to specific cancer subtypes or clinical settings. Furthermore, while cancer care in Turkey involves multiple clinical disciplines, including medical oncologists, radiation oncologists, surgical oncologists, and cardiologists, the extent to which these specialties align in their approach to CAT management has not been systematically evaluated. Addressing this knowledge gap is of critical importance, as suboptimal VTE management in cancer patients directly translates into preventable morbidity and mortality. A multidisciplinary assessment of current practice patterns is an essential first step toward identifying specific areas where targeted interventions, such as educational programs, clinical decision support tools, and nationally adapted guidelines, can improve patient outcomes.

The present study was designed to assess the degree to which current international guideline recommendations for CAT management are adopted by practicing clinicians in the Turkish healthcare environment, where differences in infrastructure, training, and resource availability may influence clinical practice. Specifically, we aimed to (1) assess clinician approaches to perioperative, inpatient, and ambulatory VTE prophylaxis; (2) evaluate the utilization of validated VTE risk assessment tools; (3) characterize anticoagulant preferences for acute VTE treatment and long-term secondary thromboprophylaxis; (4) examine management strategies for special clinical scenarios (brain tumors, renal impairment, thrombocytopenia, and pregnancy); and (5) identify self-perceived educational needs among participating clinicians. By comparing responses between oncology and cardiology specialists, this study additionally seeks to elucidate potential interdisciplinary differences in CAT management approaches.

## 2. Methods

### 2.1. Study Design

This was a cross-sectional, descriptive survey study targeting oncology and cardiology specialists practicing across Turkey. The study was approved by the Dokuz Eylul University Non-Interventional Research Ethics Committee (Protocol number: 2023/04-44, Date: 15 February 2023). Data collection was conducted between September 2024 and December 2024. All participants provided informed consent electronically prior to completing the survey. The study was conducted in accordance with the principles of the Declaration of Helsinki and Turkish regulations on the protection of personal data.

### 2.2. Study Population

The target population comprised physicians actively involved in the clinical management of cancer patients in Turkey, including medical oncologists, radiation oncologists, surgical oncologists, and cardiologists. These specialties were selected based on their direct roles in CAT management: oncology specialists predominantly contribute to primary VTE prevention during cancer treatment, while cardiologists are frequently consulted for acute VTE treatment and long-term secondary thromboprophylaxis. Participants were recruited through a combination of multiple channels to maximize reach across the target population. The survey link was disseminated via professional medical associations (including oncology and cardiology societies), institutional email networks, social media platforms and professional messaging groups, and personal professional networks of the research team. Additionally, the survey was promoted at national scientific congresses and meetings during the data collection period. A convenience sampling approach was employed, whereby all eligible physicians who accessed the survey link and provided informed consent were included. No geographical restrictions were imposed, and participation was voluntary and anonymous.

No formal sample size calculation was performed, as the study was designed as a descriptive assessment of current practice patterns rather than a hypothesis-testing investigation. A convenience sampling approach was employed with the goal of achieving a representative sample from the target population. While the final sample of 84 respondents is relatively modest, it is comparable to similar survey-based studies evaluating guideline adherence among specialists in this field [22]. However, a post-hoc power analysis based on a two-sided chi-square test for comparing two independent proportions (α = 0.05, power = 0.80) indicated that the achieved sample of 84 participants (50 cardiologists, 34 oncologists) provides adequate statistical power to detect absolute differences of approximately 25–30 percentage points between the two groups. For comparative analyses between groups, the sample provides 80% statistical power to detect medium effect sizes (Cohen’s w ≥ 0.31) at the 0.05 significance level.

### 2.3. Survey Instrument

The survey instrument was developed as a structured, case-based questionnaire comprising 21 multiple-choice questions (Supplementary File S1). Questions were designed in alignment with the most current guidelines available at the time of questionnaire development, namely the ESMO 2023 and ITAC 2022 clinical practice guidelines, and covered five major domains: (1) perioperative VTE prophylaxis in cancer surgery; (2) VTE prophylaxis in hospitalized, immobile cancer patients; (3) VTE risk assessment and primary prophylaxis in ambulatory patients receiving systemic anticancer therapy; (4) acute VTE treatment and long-term secondary thromboprophylaxis; and (5) management of VTE in special clinical scenarios including brain tumors, severe renal impairment (creatinine clearance < 30 mL/min), thrombocytopenia, and pregnancy. An additional question assessed participants’ self-perceived adequacy of their knowledge regarding CAT management. Prior to full deployment, the questionnaire was piloted among a small group of physicians to assess the clarity, comprehensibility, and appropriateness of the items, and minor revisions were made based on their feedback. The questionnaire was distributed electronically via SurveyMonkey (https://www.surveymonkey.com, access on 5 May 2026). No question skipping was permitted.

### 2.4. Data Collection and Analysis

The survey was distributed electronically between September 2024 and December 2024, and responses were collected on a rolling basis until the target sample was achieved. Responses were coded and recorded in a Microsoft Excel database. Descriptive statistics were presented as frequencies and percentages. Participants were dichotomized into two groups based on specialty: (1) the oncology group (medical oncologists, radiation oncologists, and surgical oncologists) and (2) the cardiology group. Comparative analyses between the two groups were performed using Pearson’s chi-square test or Fisher’s exact test, as appropriate. Fisher’s exact test, also known as the Freeman–Halton test, was used when expected cell frequencies were below 5. All statistical analyses were performed using IBM SPSS Statistics version 30.0 (IBM Corp., Armonk, NY, USA). A two-sided *p*-value of <0.05 was considered statistically significant. A *p*-value of <0.05 was considered statistically significant.

## 3. Results

### 3.1. Participant Characteristics

A total of 84 physicians completed the survey. Of these, 34 (40.5%) were oncology specialists (medical oncologists, radiation oncologists, or surgical oncologists) and 50 (59.5%) were cardiologists.

### 3.2. Perioperative VTE Prophylaxis

Regarding VTE prophylaxis prior to cancer surgery, 26.2% of respondents reported routinely initiating prophylactic low-molecular-weight heparin (LMWH), while the majority (67.9%) individualized prophylaxis decisions based on patient, disease, and surgical characteristics. A small proportion (4.8%) never initiated anticoagulant prophylaxis. When the decision to initiate perioperative anticoagulant prophylaxis was made, 57.8% adjusted the duration according to surgical type (7–10 days versus 4 weeks), reflecting an individualized approach consistent with guideline recommendations. Notably, 25.3% reported not initiating anticoagulation preoperatively or limiting it to the postoperative period. These findings are largely concordant with ESMO 2023 and ITAC 2022 recommendations, which advocate risk-stratified perioperative thromboprophylaxis with LMWH as the preferred agent and extended prophylaxis (up to 4 weeks) for high-risk abdominal or pelvic cancer surgery [13,14].

### 3.3. Inpatient VTE Prophylaxis

For hospitalized cancer patients with restricted mobility, 66.7% of respondents reported initiating VTE prophylaxis unless contraindicated, while 33.3% individualized the decision based on cancer type and stage. No respondent selected the options of never providing prophylaxis or using low-dose aspirin alone. Regarding anticoagulant choice, LMWH was the predominant agent (69.1%), with an additional 29.8% prescribing LMWH or unfractionated heparin (UFH) combined with compression stockings. This practice is consistent with current guideline recommendations, which uniformly endorse pharmacological VTE prophylaxis with LMWH for hospitalized cancer patients with reduced mobility in the absence of contraindications [13,14,15,16].

### 3.4. Ambulatory VTE Risk Assessment

Among the most notable findings, 47.0% of respondents reported not performing any VTE risk assessment for ambulatory patients receiving systemic anticancer therapy, while only 33.7% performed risk assessment for all patients. When asked about specific risk scoring tools, 64.6% did not use any validated scoring system. Among those who did, the COMPASS-CAT score (15.9%) was slightly more frequently used than the Khorana score (13.4%), followed by the Vienna-CATS score (6.1%). These findings indicate a significant departure from current guidelines, which consistently recommend systematic VTE risk assessment of all ambulatory cancer patients receiving systemic therapy, preferably using validated tools such as the Khorana score [13,14,15,16].

### 3.5. Patient Education on VTE

Regarding patient education about VTE risk, symptoms, and signs, only 28.9% reported routinely informing all patients. The majority provided selective information based on perceived risk level: 39.8% informed patients with moderate-to-high risk, 16.9% informed only high-risk patients, and 14.5% did not provide any VTE-related education. Although specific recommendations on patient education vary across guidelines, ESMO 2023 and ITAC 2022 emphasize the importance of informing all cancer patients about VTE risk and symptoms as part of comprehensive cancer care [13,14].

### 3.6. Primary Prophylaxis in Ambulatory Patients

For primary VTE prophylaxis in ambulatory patients, 34.9% administered prophylaxis based on formal risk scoring, while an equal proportion (34.9%) prescribed prophylaxis based on clinical judgment without formal risk assessment. Importantly, 26.5% did not provide primary prophylaxis to any ambulatory patient. When primary prophylaxis was initiated, LMWH was the most frequently selected agent (65.9%), followed by rivaroxaban (18.3%), apixaban (8.5%), and other antithrombotic agents (7.3%). Current guidelines recommend primary thromboprophylaxis in high-risk ambulatory cancer patients identified through validated risk assessment tools, with LMWH and DOACs (rivaroxaban and apixaban) as recommended agents based on the CASSINI and AVERT trials [11,12,13,14].

### 3.7. Acute VTE Treatment

In the acute phase (first 10 days) of cancer-associated VTE, LMWH was the preferred initial anticoagulant for 72.6% of respondents, while 13.1% selected the treatment based on individual patient and disease characteristics. DOACs and UFH were each chosen by 7.1%. This preference aligns with ESMO 2023 and ITAC 2022 recommendations, which position LMWH as a first-line agent for initial treatment of cancer-associated VTE, while also recognizing DOACs as an acceptable alternative in patients without high bleeding risk or gastrointestinal/genitourinary tract involvement [13,14].

### 3.8. Long-Term Secondary Thromboprophylaxis

For long-term secondary thromboprophylaxis beyond the acute phase, 55.9% individualized the duration based on patient and disease factors, 36.9% prescribed a fixed 6-month course, and 6.0% limited it to 3 months. Regarding agent selection for long-term secondary thromboprophylaxis, DOACs emerged as the most frequently preferred option (42.2%), followed by LMWH (31.3%), with 25.3% selecting the agent based on clinical circumstances. The criteria for extending long-term secondary thromboprophylaxis to 12 months were predominantly guided by the persistence of risk factors such as active cancer or ongoing anticancer treatment (65.1%). These practices are broadly consistent with guideline recommendations, which advocate individualized duration of anticoagulation based on ongoing risk factors and recognize both LMWH and DOACs as appropriate agents for long-term secondary thromboprophylaxis, with a minimum duration of 6 months [13,14,15,16].

### 3.9. Recurrent VTE Under Anticoagulation

For recurrent VTE developing under anticoagulation, 34.6% switched between LMWH and DOACs (i.e., switching to DOACs if the event occurred under LMWH and vice versa), 28.4% increased the LMWH dose or changed the DOAC agent, 27.2% switched to warfarin regardless of the prior agent, and 9.9% switched to UFH. Guidelines recommend switching between anticoagulant classes (LMWH to DOAC or vice versa) or increasing the LMWH dose in cases of recurrent VTE under anticoagulation, which is consistent with the approaches reported by the majority of respondents [13,14].

### 3.10. Special Clinical Scenarios

In patients with brain tumors who developed VTE, 72.8% preferred LMWH, 12.4% preferred DOACs, 8.6% chose UFH, and 6.2% would never administer anticoagulation. For brain tumor patients undergoing neurosurgery, 69.5% provided LMWH prophylaxis, while 22.0% avoided pharmacological prophylaxis entirely. In severe renal impairment (creatinine clearance < 30 mL/min), LMWH remained the predominant choice (64.6%), followed by UFH (20.7%) and DOACs (13.4%). For thrombocytopenic patients with VTE, 48.2% administered standard-dose anticoagulation when platelet count exceeded 50,000/mL, and 42.2% used dose-reduced anticoagulation under the same threshold. In pregnant cancer patients with VTE, LMWH was overwhelmingly preferred (78.3%), followed by UFH (16.9%). These preferences are largely aligned with guideline recommendations: LMWH is the preferred agent for VTE in brain tumor patients and during pregnancy, while dose-adjusted UFH or LMWH is recommended in severe renal impairment. For thrombocytopenic patients, guidelines recommend full-dose anticoagulation when platelet counts exceed 50,000/μL, with dose reduction or transfusion support below this threshold [13,14,15,16,18]. Notably, the 13.4% of respondents who would use DOACs in severe renal impairment represents a potential area of concern, as this practice is not supported by current guidelines.

### 3.11. Self-Perceived Knowledge Adequacy

Remarkably, 94.1% of respondents expressed a desire to update and improve their knowledge regarding VTE prevention and treatment in cancer patients, while only 5.9% considered their current knowledge adequate and up-to-date.

### 3.12. Comparison Between Oncologists and Cardiologists

Comparative analysis between the oncology and cardiology groups revealed no statistically significant differences across any of the survey items (all *p* > 0.05). Response distributions were remarkably similar between the two specialties for perioperative prophylaxis, inpatient management, risk assessment practices, acute treatment, long-term secondary thromboprophylaxis, and management of special scenarios. Both groups demonstrated comparable rates of knowledge update desire (94.0% cardiologists vs. 94.1% oncologists, *p* = 0.99). The detailed comparison is presented in Table 1.

## 4. Discussion

This cross-sectional survey provides the first comprehensive, multidisciplinary assessment of oncologist and cardiologist adherence to international VTE prevention and treatment guidelines in cancer patients in Turkey. Our findings reveal a complex landscape: while overall awareness of CAT as a clinical entity appears robust, and several domains of clinical practice demonstrate substantial alignment with guideline recommendations, significant gaps persist—particularly in ambulatory VTE risk assessment, systematic utilization of validated risk scoring tools, and primary prophylaxis decision-making.

One of the most reassuring findings of this study is the high level of guideline concordance observed in perioperative and inpatient VTE prophylaxis. The majority of respondents demonstrated awareness that cancer surgery confers elevated VTE risk and reported individualizing prophylaxis decisions based on patient, disease, and surgical characteristics. This approach aligns well with ESMO 2023, ITAC 2022, ASCO 2020, and BSH 2024 recommendations, which emphasize risk-stratified perioperative thromboprophylaxis with LMWH as the preferred agent [13,14,15,16,17,18]. The predominance of LMWH as the preferred prophylactic agent (69.1% for inpatient prophylaxis) further corroborates guideline adherence in this domain. Similarly, the near-universal recognition that hospitalized, immobile cancer patients warrant pharmacological VTE prophylaxis reflects adequate integration of established guideline principles into clinical practice.

In contrast, the management of ambulatory cancer patients represents the most concerning area of practice divergence from guideline recommendations. Nearly half (47.0%) of respondents reported not performing any VTE risk assessment in ambulatory patients receiving systemic anticancer therapy, and almost two-thirds (64.6%) did not use any validated risk scoring system. This finding is particularly noteworthy given that international guidelines consistently recommend risk stratification of ambulatory patients using validated tools—most notably the Khorana score—to identify candidates for primary thromboprophylaxis [13,14,15,16,17,18]. The Khorana score, while imperfect in its predictive accuracy, remains the most widely validated and guideline-endorsed risk assessment model for ambulatory cancer patients [23]. The underutilization of this and other validated tools observed in our study suggests a significant implementation gap that may result in missed opportunities for thromboprophylaxis in high-risk ambulatory patients.

These findings are consistent with international literature demonstrating suboptimal VTE risk assessment practices in ambulatory cancer patients. A previous Turkish survey by Kandemir et al. similarly identified limited use of risk scoring systems among medical oncologists, though that study was conducted in 2018—prior to the publication of several landmark trials and updated guidelines [22]. The present study extends these observations by including cardiologists, updating the assessment to reflect the post-DOAC era, and demonstrating that the risk assessment gap persists despite the evolving evidence base and increased VTE awareness following the COVID-19 pandemic.

The treatment of acute cancer-associated VTE showed strong guideline adherence, with LMWH selected as the initial anticoagulant by 72.6% of respondents. This finding is consistent with ESMO 2023 and ITAC 2022 recommendations, which position LMWH as the first-line agent for acute CAT management [13,14]. Notably, DOACs have emerged as the preferred agent for long-term secondary thromboprophylaxis (42.2%), reflecting the growing clinical adoption of these agents following pivotal trials such as Hokusai VTE-Cancer, SELECT-D, CARAVAGGIO, and others [24,25,26]. The transition from LMWH-dominant acute treatment to DOAC-favored long-term secondary thromboprophylaxis mirrors the evolving guideline landscape and suggests that clinicians are appropriately differentiating between the acute and maintenance phases of CAT management.

The management of special clinical scenarios revealed generally appropriate clinical judgment, with LMWH consistently preferred in high-risk situations such as brain tumors (72.8%), severe renal impairment (64.6%), and pregnancy (78.3%). These preferences align with guideline recommendations that favor LMWH in scenarios where DOAC pharmacokinetics may be unpredictable or where bleeding risk is elevated [13,14,15,16,17,18]. However, the observation that 13.4% of respondents would use DOACs in patients with creatinine clearance below 30 mL/min raises concerns about potential overuse in this population, as most DOACs are at least partially renally cleared and their safety in severe renal impairment is not well established.

Perhaps the most striking finding of this study is that 94.1% of participating clinicians acknowledged that their knowledge of CAT management requires updating. This near-universal self-assessment transcends specialty boundaries, with virtually identical rates observed among cardiologists (94.0%) and oncologists (94.1%). This finding carries important implications for medical education and continuing professional development. It suggests that despite adequate baseline awareness, the rapidly evolving nature of CAT management—with new therapeutic agents, updated guidelines, and emerging evidence from ongoing trials—creates a persistent educational gap that current training and professional development structures may not adequately address.

The absence of statistically significant differences between oncologists and cardiologists across all survey items is itself a noteworthy finding. While one might hypothesize that specialty-specific training and clinical exposure would produce divergent approaches to CAT management, our data suggest remarkable interdisciplinary alignment. This alignment may reflect shared exposure to major clinical guidelines, overlapping conference attendance, and the increasingly collaborative nature of cancer care. However, it could also indicate that both groups face similar limitations in guideline implementation, pointing to systemic rather than discipline-specific barriers.

Several implications for clinical practice emerge from these findings. First, the integration of validated VTE risk assessment tools into electronic health records and clinical decision support systems could substantially improve risk stratification practices in ambulatory settings. Automated alerts triggered by patient data entry, indicating elevated CAT risk, could bridge the gap between guideline recommendations and clinical implementation. In this context, multimodal assessment approaches incorporating imaging-based cardiovascular evaluation tools may further complement clinical risk scoring systems in cardio-oncology settings [27]. Second, structured, multidisciplinary educational programs focusing specifically on CAT management should be developed, with particular emphasis on ambulatory risk assessment, DOAC utilization in appropriate patient populations, and management of complex clinical scenarios. Third, the establishment of national-level, standardized operating procedures for CAT management, adapted from international guidelines to reflect Turkish healthcare realities, could promote practice homogeneity and quality improvement.

From a cardio-oncology perspective, this study reinforces the notion that CAT management represents a core competency of the emerging cardio-oncology discipline, extending beyond its traditional focus on cardiotoxicity assessment. The active involvement of cardiologists in CAT management, as evidenced by their comparable awareness and practice patterns, supports the integration of thromboembolism modules into cardio-oncology training curricula.

Several limitations should be acknowledged. First, the relatively modest sample size (*n* = 84) may limit the generalizability of our findings to the broader physician population in Turkey. No formal sample size calculation was performed given the descriptive nature of the study and the absence of a predefined primary hypothesis. Nevertheless, the sample size is comparable to previously published survey studies evaluating CAT guideline adherence [22], and the consistency of responses across specialty groups suggests that the findings capture meaningful practice patterns. Future studies with larger, probability-based samples are warranted to confirm and extend these observations. Second, the survey-based, cross-sectional design relies on self-reported practices, which may not fully reflect actual clinical behavior. Social desirability bias may have influenced responses, potentially leading to overestimation of guideline-concordant practices. Physicians may report what they believe to be the correct approach rather than what they actually do in clinical practice. Future studies incorporating medical record reviews, prescription audits, or prospective observational designs would provide more accurate assessments of real-world guideline adherence. Third, only oncology and cardiology specialists were included; other disciplines involved in cancer care (intensivists, internists, vascular surgeons, interventional radiologists) were not represented. Fourth, the questionnaire was intentionally limited in length to optimize completion rates, precluding detailed assessment of certain clinical scenarios, particularly the management of hemodynamically unstable pulmonary embolism. Additionally, although the questionnaire was piloted for clarity and was directly derived from international guideline recommendations to ensure content validity, formal psychometric validation including test–retest reliability and internal consistency assessment was not performed. Future studies should consider employing validated instruments. Furthermore, the response rate could not be determined, as the survey was disseminated through multiple open channels and the total number of physicians who received or viewed the survey link is unknown. Additionally, the geographic distribution of respondents across Turkey was not assessed, which may introduce regional clustering bias and limits our ability to evaluate potential regional variations in practice patterns. Despite these limitations, this study provides a valuable baseline assessment and serves as a foundation for future, more comprehensive investigations.

## 5. Conclusions

This study demonstrates that oncologists and cardiologists in Turkey maintain general awareness of cancer-associated thrombosis and exhibit substantial alignment with international guideline recommendations in several clinical domains, particularly perioperative and inpatient VTE prophylaxis and acute VTE treatment. However, significant gaps persist in ambulatory VTE risk assessment and primary prophylaxis, where the systematic use of validated risk scoring tools remains limited and clinical decision-making is heterogeneous. The near-universal acknowledgment of knowledge deficiency among participating clinicians represents both a challenge and an opportunity—a challenge in that it reflects the complexity and rapid evolution of CAT management, and an opportunity in that it signals a receptive audience for targeted educational interventions. The absence of significant interdisciplinary differences suggests that barriers to optimal CAT management are systemic rather than specialty-specific and that improvement strategies should be designed to engage all relevant clinical disciplines. Future efforts should focus on (1) developing structured, multidisciplinary education programs emphasizing evidence-based CAT management; (2) integrating validated risk assessment tools into clinical workflows through electronic decision support systems; (3) establishing national clinical practice guidelines adapted to the Turkish healthcare context; and (4) conducting larger-scale, multi-center studies to generate Turkey-specific epidemiological and outcome data. These steps, taken collectively, have the potential to significantly improve the quality of CAT prevention and treatment, ultimately benefiting patient outcomes.

## Figures and Tables

**Table 1:** Comparison of survey responses between cardiologists and oncologists. VTE, venous thromboembolism; LMWH, low-molecular-weight heparin; DOAC, direct oral anticoagulant; CI, contraindication; RF, risk factor; std, standard.

Survey Domain	Total (*n* = 84)	Cardiologists (*n* = 50)	Oncologists (*n* = 34)	*p*-Value *
Preoperative VTE prophylaxis approach	67.9% individualized	66.0% individualized	70.6% individualized	0.51
Perioperative anticoagulation strategy	57.8% surgery-type based	60.0% surgery-type based	54.5% surgery-type based	0.18
Inpatient VTE prophylaxis	66.7% if no CI	74.0% if no CI	55.9% if no CI	0.10
Inpatient anticoagulant choice	69.1% LMWH	68.0% LMWH	70.6% LMWH	0.53
Ambulatory VTE risk scoring	33.7% all patients	36.7% all patients	29.4% all patients	0.89
Risk scoring tool used	64.6% none	67.3% none	60.6% none	0.40
Acute VTE initial treatment	72.6% LMWH	74.0% LMWH	70.6% LMWH	0.39
Long-term secondary thromboprophylaxis duration	55.9% individualized	56.0% individualized	55.9% individualized	0.92
Long-term secondary thromboprophylaxis agent	42.2% DOAC	42.0% DOAC	41.2% DOAC	0.88
Extension to 12 months	65.1% if RF persist	64.0% if RF persist	64.7% if RF persist	0.99
Recurrent VTE management	34.6% LMWH/DOAC switch	34.0% LMWH/DOAC switch	32.4% LMWH/DOAC switch	0.71
Brain tumor + VTE	72.8% LMWH	72.0% LMWH	67.6% LMWH	0.62
Brain tumor + neurosurgery	69.5% LMWH	70.0% LMWH	64.7% LMWH	0.58
Severe renal impairment + VTE	64.6% LMWH	64.0% LMWH	61.8% LMWH	0.83
Thrombocytopenia + VTE	48.2% std dose if >50 K	48.0% std dose if >50 K	47.1% std dose if >50 K	0.91
Pregnancy + VTE	78.3% LMWH	78.0% LMWH	76.5% LMWH	0.88
Knowledge update desire	94.1% yes	94.0% yes	94.1% yes	0.99

* Chi-square test or Fisher’s exact test, as appropriate.

## Data Availability

The datasets used and/or analyzed in this study are available upon reasonable request from the corresponding author.

## Supplementary Materials

The following supporting information can be downloaded at: https://www.mdpi.com/article/10.3390/jcm15124504/s1, Supplementary File S1: Survey Questionnaire.
