# Supplementary material for: Adherence of Oncologists and Cardiologists to Venous Thromboembolic Disease Prevention and Treatment Guidelines in Cancer Patients: A Cross-Sectional Survey from Turkey
Source: J Clin Med. 2026 Jun 10;15(12):4504. doi: 10.3390/jcm15124504 (PMC13300970; doi:10.3390/jcm15124504)
Supplement: Supplementary file 1 [file jcm-15-04504-s001.zip › jcm-4330689-supplementary.pdf]

Supplementary File S1

Survey Questionnaire

Informed Consent Statement

Dear Physician,

This survey has been designed within the scope of a doctoral thesis to understand the prevention and treatment strategies of oncology and cardiology specialists regarding venous thromboembolic diseases, including deep vein thrombosis and pulmonary embolism, in cancer patients in their daily clinical practice.

Participation in this survey is entirely voluntary. If you agree to participate, no identifying information will be collected; the only personal data requested will be your specialty, years of experience, and type of healthcare institution where you practice.

This project has received approval from the Dokuz Eylul University Non-Interventional Research Ethics Committee (Protocol number: 2023/04-44, Date: 15 February 2023).

No payment will be made to you or your institution for participation in this survey. The survey results will be published in the doctoral thesis and in related scientific outputs (congress presentations, articles, etc.).

This survey is not intended to measure your level of knowledge on the subject, nor does it contain any guidance aimed at changing your daily clinical practice.

The survey consists of 21 questions and takes approximately 15 minutes to complete.

SECTION I: PRIMARY PREVENTION OF VENOUS THROMBOEMBOLISM

D1: What is your area of expertise?

(a) Medical oncology.
(b) Radiation oncology
(c) Oncological surgery.
(d) Cardiology.

Question 1

To prevent venous thromboembolism (VTE) before cancer surgery, I:

(a) Routinely initiate prophylactic low-molecular-weight heparin (LMWH).
(b) Routinely initiate prophylactic unfractionated heparin (UFH).
(c) Do not initiate prophylaxis for all patients; I decide based on patient, disease, and surgical characteristics.
(d) Never initiate any anticoagulant therapy.

Question 2

If I decide to initiate anticoagulant therapy (ACT) for VTE prevention before cancer surgery, I:

(a) Start before the operation and discontinue after 7–10 days.

(b) Start before the operation and discontinue after 4 weeks.

(c) Start before the operation and continue for 7–10 days or 4 weeks depending on the type of surgery.

(d) Do not initiate anticoagulant therapy preoperatively, or administer it only in the postoperative period.

Question 3

For my hospitalized cancer patient with restricted mobility, I:

(a) Never provide VTE prophylaxis.

(b) Decide on VTE prophylaxis based on individual patient/disease characteristics (cancer type, stage, etc.).

(c) Initiate VTE prophylaxis unless there is a contraindication to anticoagulant therapy.

(d) Do not administer anticoagulant therapy but prescribe low-dose aspirin.

Question 4

If I decide to initiate anticoagulant therapy for VTE prophylaxis in my hospitalized cancer patient with restricted mobility, I:

(a) Initiate low-molecular-weight heparin (LMWH).

(b) Initiate unfractionated heparin (UFH).

(c) Initiate LMWH or UFH and additionally apply compression stockings.

(d) Initiate LMWH or UFH without recommending compression stockings.

Question 5

For my ambulatory patient receiving systemic anticancer therapy, I perform VTE risk assessment using a risk scoring method:

(a) Not at all.

(b) Only for specific cancer types.

(c) Only for patients above a certain age.

(d) For all my patients.

Question 6

For VTE risk assessment in my ambulatory patient receiving systemic anticancer therapy, I use:

(a) No risk scoring method.

(b) The Khorana Risk Score.

(c) The COMPASS-CAT Score.

(d) The Vienna-CATS Score.

Question 7

Regarding informing my ambulatory patients receiving systemic anticancer therapy about VTE risk, symptoms, and signs, I:

(a) Routinely inform all my patients.

(b) Only inform patients with moderate-to-high VTE risk.

(c) Only inform patients with high VTE risk.

(d) Do not provide any VTE-related information to any of my patients.

Question 8

Regarding primary prophylaxis with anticoagulant therapy for my ambulatory patient receiving systemic anticancer therapy, I:

(a) Provide prophylaxis to all my patients.

(b) Perform risk scoring and provide prophylaxis only to high-risk patients.

(c) Do not perform risk scoring but provide prophylaxis to patients I consider to be at high risk.

(d) Do not provide primary prophylaxis to any of my ambulatory patients.

Question 9

If I decide to provide VTE prophylaxis (primary prophylaxis) to my ambulatory patient receiving systemic anticancer therapy, I prescribe:

(a) LMWH (e.g., enoxaparin 1 mg/kg) for six months.

(b) Apixaban for six months.

(c) Rivaroxaban for six months.

(d) A different antithrombotic agent (aspirin, warfarin, etc.).

SECTION II: TREATMENT OF VENOUS THROMBOEMBOLISM

Question 10

For my cancer patient who develops a venous thromboembolic event (acute DVT or acute pulmonary embolism) during the course of cancer, as the initial anticoagulant therapy in the ACUTE PHASE (first 10 days), I initiate:

(a) Low-molecular-weight heparin (LMWH).

(b) Unfractionated heparin (UFH).

(c) A direct oral anticoagulant (apixaban, rivaroxaban, or edoxaban).

(d) One of the above treatment options depending on individual patient/disease characteristics.

Question 11

For my cancer patient who develops a venous thromboembolic event, AFTER THE ACUTE PHASE (first 10 days), I provide anticoagulant prophylaxis (secondary prophylaxis) for:

(a) 3 months.

(b) 6 months.

(c) 12 months.

(d) A duration determined based on individual patient/disease characteristics.

Question 12

For secondary prophylaxis after the acute phase in my cancer patient with VTE, I prescribe:

(a) Low-molecular-weight heparin (LMWH).

(b) Unfractionated heparin (UFH).

(c) A direct oral anticoagulant (apixaban, edoxaban, rivaroxaban).

(d) One of the above treatment options depending on individual patient/disease characteristics.

Question 13

I extend the duration of secondary prophylaxis up to 12 months in my cancer patient with VTE when:

(a) I provide secondary prophylaxis up to 12 months for all my patients.

(b) Only when VTE risk factors (cancer, anticancer treatment, etc.) persist.

(c) Only when VTE recurs during the initial 6 months of secondary prophylaxis.

(d) I never extend secondary prophylaxis duration up to 12 months under any circumstances.

Question 14

For my cancer patient who develops recurrent VTE while on anticoagulant therapy, I:

(a) Increase the LMWH dose if recurrence occurred under LMWH; switch the DOAC agent if recurrence occurred under a DOAC.

(b) Switch to a direct oral anticoagulant (apixaban, edoxaban, rivaroxaban) if recurrence occurred under LMWH; switch to LMWH if recurrence occurred under a DOAC.

(c) Switch to unfractionated heparin (UFH) regardless of whether recurrence occurred under a DOAC or LMWH.

(d) Switch to warfarin regardless of whether recurrence occurred under a DOAC or LMWH.

Question 15

For my patient with a brain tumor who develops VTE during the course of disease, I:

(a) Never administer anticoagulant therapy.

(b) Prefer low-molecular-weight heparin (LMWH).

(c) Prefer unfractionated heparin (UFH).

(d) Prefer a direct oral anticoagulant (apixaban, edoxaban, rivaroxaban).

Question 16

For VTE prophylaxis in my patient with a brain tumor who is planned for neurosurgery, I:

(a) Never provide anticoagulant prophylaxis.
(b) Prefer low-molecular-weight heparin (LMWH).
(c) Prefer unfractionated heparin (UFH).
(d) Prefer a direct oral anticoagulant (apixaban, edoxaban, rivaroxaban).

Question 17

For my patient with severe renal impairment (creatinine clearance <30 mL/min) who develops VTE during the course of cancer, I:

(a) Never administer anticoagulant therapy.
(b) Prefer low-molecular-weight heparin (LMWH).
(c) Prefer unfractionated heparin (UFH).
(d) Prefer a direct oral anticoagulant (apixaban, edoxaban, rivaroxaban).

Question 18

For my patient with thrombocytopenia who develops VTE during the course of cancer, I:

(a) Never administer anticoagulant therapy.
(b) Administer standard-dose anticoagulant therapy if platelet count is >50,000/mL.
(c) Administer dose-reduced anticoagulant therapy if platelet count is >50,000/mL.
(d) Administer anticoagulant therapy regardless of platelet count.

Question 19

For my pregnant patient who develops VTE during the course of cancer, I:

(a) Never administer anticoagulant therapy.
(b) Prefer low-molecular-weight heparin (LMWH).
(c) Prefer unfractionated heparin (UFH).
(d) Prefer a direct oral anticoagulant (apixaban, edoxaban, rivaroxaban).

Question 20

Regarding my knowledge on the prevention and treatment of venous thromboembolic diseases in cancer patients:

(a) I consider my current knowledge to be sufficient and up-to-date.
(b) I would like to improve and/or update my current knowledge.
